# A Health and Nutritional Evaluation of Changes in Agriculture in the Past Quarter Century in British Columbia: Implications for Food Security

**DOI:** 10.3390/ijerph7062653

**Published:** 2010-06-17

**Authors:** Aleck Ostry, Kathryn Morrison

**Affiliations:** 1 Social Sciences, Department of Geography, University of Victoria, Victoria, V8W 3R4, Canada; 2 Population Health Lab, Spatial Pattern & Analysis Research Lab, Graduate Studies, Department of Geography, University of Victoria, Victoria, V8W 3R4, Canada; E-Mail: ktm@uvic.ca

**Keywords:** food security, nutrition policy, agriculture, British Columbia, health audit

## Abstract

This paper describes change in local food production in British Columbia with a focus on changes in the production of foods recommended for increased consumption by nutritionists. We determine, in one of the most productive agricultural provinces in Canada, whether secular trends in agricultural land use and food production, over the past quarter century, have resulted in increased production of foods recommended by nutritionists as more healthy and nutritious. In particular we are concerned with estimating the extent to which changes in agriculture and food production are congruent with official nutrition advice to avoid less healthy foods and to consume more vegetables, fruit, and whole grains. We demonstrate, using regularly collected agricultural census data, in spite of nutritionists’ advocacy for improved access to locally produced fruits, vegetables, and grains, since 1986, that BC agriculture is moving firmly in the opposite direction with greater production of animal fats, and hay and grain for animal feed and much reduced production of traditional fruits, vegetables, and grains designed mainly for human consumption. While nutritionists advise us to increase consumption especially of whole grains, vegetables and fruit, local production capacity of these foods in BC has decreased markedly between 1986 and 2006. In conclusion, there is a structural disconnect between the kinds of foods produced in BC and the nutritional needs of the population.

## 1. Introduction

There have been rapid increases in both obesity and sedentary behaviours in the population of most developed nations over the past quarter century [1–3]. The increased rates of obesity, in particular, have, in turn, been strongly linked to growing prevalence of chronic diet-related illnesses especially diabetes and coronary heart disease and especially among children [4–6]. Policy makers are increasingly aware that interventions to reduce current levels or prevent future increases in obesity in order to reduce chronic diet related illness will need to be applied across the life-course and will ultimately require major structural and cultural changes in society that shift working, leisure, and family environments in order to reduce their obesogenicity [7–10].

These interventions will likely involve a range of traditional health promotion programs targeted at individuals and designed to shift dietary and physical activity habits as well as programs and policies to change basic social infrastructure and cultural attitudes to create an environment that makes it much easier, than at present, to eat healthily and to exercise [11–13]. Systems of agriculture and food production along the continuum from food production to consumption will have to change too [12,14–16]. In particular, it will be increasingly important to ensure that we produce healthier than unhealthy foods, that we produce these foods in adequate quantities and, that they are well priced and of high enough quality to feed local populations [17–19].

While some healthy foods will never be produced in certain locations simply because of deficiencies in climate, soil, or access to land, and will therefore require importation, it is essential to the promotion of local food security, to determine the capacity of local agricultural and food production systems to supply healthy and affordable foods for local populations. In this paper we develop a “health audit” or nutritional assessment of local food production in the province of British Columbia, Canada, in order to ascertain the extent to which foods produced in the province align with broad recommendations for healthy eating promulgated by Canadian nutritional authorities. We also determine whether or not local food production capacity for these (as well as for less healthy foods) can in fact meet the needs of the province’s population of about 5 million. Our investigation has an historical component as we track changes in the production of basic foods in the province from 1986 through to 2006. We take an historical approach to demonstrate trends in food production over the medium term as these provide information on the character of changes in food production that cannot be obtained from a strictly cross-sectional approach, and demonstrate the direction and momentum of this change as this may foreshadow the future character and potential for local food production capacity over the short and medium term, assuming no major or sudden changes in markets or policy.

We are conducting this investigation in Canada’s western most province, British Columbia (BC) because it has (1) some of the most productive agricultural land in the world, (2) a unique agricultural land protection regime (the Agricultural Land Reserve, (3) a motivated government, especially in the ministries of health and agriculture, which have over the past several years become heavily involved in funding novel food security programs, (4) provincial medical health officer and nutritionist leadership (at the provincial government and the regional health authority level), focused on promoting food security as fundamental to preventing chronic diet-related illness in the population, and (5) a strong community-based food security movement.

These features of the province of BC arguably lend themselves to the development of successful partnerships to move agricultural and food systems in the province to increased production of more, rather than less, healthy foods. BC is therefore an interesting case study as it has many features in place that should, in theory, have helped move the province towards greater local production of healthy foods. Finally, BC is the birthplace of the 100 mile diet concept (*i.e.*, the notion that consumers should, in an ideally food secure world, be able to obtain most of their dietary requirements, year round, from sources located within a 100 mile radius of their home). In this paper, we put this idea to an empirical test by asking the linked questions “how much and what types of foods were produced by the province’s agricultural system a quarter century ago” and “how much and what types of foods are now produced”. By analyzing these trends in basic food production we demonstrate how locally food secure the province has been, how locally food secure it is at present, and how far and how policy makers need to move to encourage change in current systems of food production in the province to establish food practices and systems that are more congruent with modern nutritional advice on healthy eating.

## 2. Methods

We have obtained regularly collected data on food production of the most common foods produced in BC, from Statistics Canada, for the period from 1986 to 2006. This allows us to track the extent of change in production of fruit, vegetables, meat, meat products, dairy products and grains over this time. There are two sets of data used in this paper, both obtained from Statistics Canada. The primary dataset is the Census of Agriculture, performed every 5 years in Canada. The Census measures the area of land planted in fruit, vegetables, oil seeds, grains, and other crops and also counts the number of animals. And, in order to convert the area of land planted in various crops into quantities of these products and to convert the number of animals into quantity produced, we have utilized regularly conducted Statistics Canada surveys of yields for major crops and for animals over this time period. All major vegetable and fruit crops in BC were included in our calculations except pumpkins, squash, zucchini, and cherries as these were not included in the yield surveys until 2007.

In 1986 the population of BC was 3,003,621. By 2006 this had increased by 41.3% to 4,243,580. Statistics Canada publishes national food disappearance data for these years [20]. These are estimates, by major food category, of the per-capita consumption of these foods by Canadians. These are based on Canadian import, export, and production data and include estimates for waste and are therefore estimates of amounts of food that end up on the consumer’s plate. These are Canadian data and may, therefore, not accurately reflect per-capita consumption of British Columbians. We have used Canadian data for 1986 and 2006 because food consumption data for the BC population does not exist for these years. In 1999 a fairly comprehensive and representative survey of the food consumption was conducted in BC. Estimates of consumption obtained from this 1999 survey are comparable to estimates obtained from Statistics Canada’s national food disappearance data in 1999 giving us reasonable confidence that use of national, rather than BC data for 1986 and 2006, will result in reasonable estimates of food consumption patterns among British Columbians. Therefore, we have multiplied the per-capita food consumption values obtained from the national food disappearance data base for major food categories by the BC population in 1986 and 2006 to estimate consumption, by major food categories, for British Columbians. Our consumption estimates for these categories (fruit, potatoes, field vegetables, cereals, milk, meat and eggs) are presented in the Results section in Table 7.

To assess changes in provincial self-sufficiency, we require a food production estimate that is directly comparable to food consumption, in units of weight. By combining units of production (hectares of farmland or number of animals) with estimates of yield (kilograms of food produced per hectare or animal), we can estimate provincial food production. Our estimates are based on data reported in the Census of Agriculture, and therefore includes production coming only from “census farms” at least one acre in size, as defined by Statistics Canada. Our estimates therefore do not include production from very small farms and farmers who produce food for their own consumption.

## 3. Results

### 3.1. Changes in Land Use for Food Growing in BC 1986–2006

Table 1 shows that the hectarage of farmland in BC has increased by nearly 20% between 1986 and 2006 to approximately 2.8 million hectares. Land devoted to field vegetable production has decreased by 8 percent (although land devoted specifically to potatoes increased by about 8 percent) and land devoted to fruit production has increased by about 10 percent. During this time, the area under glass for greenhouse vegetable production increased by over 400%. The area of enclosed greenhouse space, located primarily in the lower mainland and Vancouver Island and devoted mainly to tomatoes, bell peppers, and cucumbers, was approximately 4 million square meters by 2006 (Table 1). Thus, while the hectarage devoted to field fruits and vegetables has remained relatively stable, the greenhouse industry has expanded rapidly.

The major trend in land use between 1986 and 2006 is the increase (approximately 500,000 hectares) in land devoted to pasturage (*i.e.*, to animal food production). Over the same time period, land planted with grain decreased by more than 40%. Specifically, land used for grain production declined from 220,356 to 128,497 hectares between 1986 and 2006. These data indicate that the major trend in land use for food production in BC between 1986 and 2006 was the growth in land used for animal production.

Given that major changes in land use have occurred for animal production, we turn in the next section to a more detailed consideration of changes in meat, dairy, and egg production in the province over this twenty year time frame.

### 3.2. Changes in Meat, Animal, & Dairy Production in BC 1986–2008

There are no data available on the extent to which the approximately 1.8 million hectares of land devoted to pastureland in BC in 2006 was used to feed dairy cows *vs.* beef cows, or other animals. However, given that cattle require more pasture land than other animals and, given that the main change in the number of animals over this twenty year period was an increase in non-dairy cattle and calves, it is likely that increase in pasturage over this time went mainly to produce more beef and/or dairy products. Two other major trends noted are the steady decline in pig production over this time and the doubling in poultry production (Table 2).

National consumption data have shown clear acceleration in chicken consumption over the past quarter century [21]. Thus, it is no surprise to see a doubling of hen and chicken numbers in BC. These huge increases in poultry production will not be visible in land use figures as most of this production is conducted at intensive factory farms [22].

Milk production has increased by nearly 30% and egg production by almost 75% in the period 1986 to 2006 (Table 3). Given that the number of dairy cows has remained relatively steady, the increase in production is due to greater milk yields per cow. Conversely, egg yield (eggs produced per hen on average) is relatively unchanged since 1986, so that the significant increase in egg production is due to the increase in laying hens in BC.

In general, livestock yields, in terms of the amount of edible meat produced per animal, have increased in a slow but steady manner (by about10%) since 1986.

In summary, cattle and calves numbers have increased in BC. The number of dairy cows has remained fairly constant. The number of hogs has decreased considerably while sheep and lambs remained fairly stable, and both form relatively small industries in BC. The size of the poultry industry has doubled in BC as has provincial egg production. The increases in pasturage are likely due to greater production of beef cattle. In the following section we track changes in the production of vegetables in BC.

### 3.3. Changes in the Production of Vegetables in BC, 1986–2006

**Figure 1.**
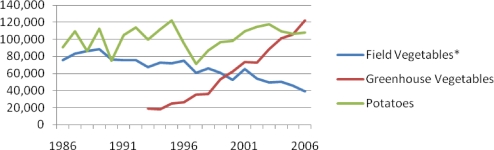
Tonnes of Vegetable Production in BC, 1986–2006. * Excluding potatoes Data source: Statistics Canada CANSIM Table 001–0013 & Greenhouse, Sod, & Nursery Industries Catalogue 22-202-XWE.

Figure 1 shows that total vegetable production in BC in 2006 was approximately 275,000 tonnes. While field grown potato production has remained fairly steady since 1986 (at about 100,000 tonnes) the production of other field grown vegetables has declined by about 50 percent to 40,000 tonnes. In contrast, in 2006, greenhouse production (mainly focused on cucumbers, tomatoes, and bell peppers and mainly for the export market), was approximately 120,000 tonnes accounting for three times the weight of field grown (non-potato) vegetable production.

Table 4 shows that mean potato production has increased by about a third since the late 1980s, whereas other field vegetable production has decreased by 40% over this time. Greenhouse production has increased by 437% since the early 1990s.

### 3.4. Change in the Production of Fruit in BC, 1986–2006

Table 5 illustrates that overall fruit production declined by about one quarter in this period with declines of over 50 percent for strawberries, and plums and prunes. Apple production, (accounting for roughly two thirds of all fruit produced in 2006) declined by about one third over this period as did the production of pears and peaches. Major gains in production were witnessed for blueberries (+245%) and grapes (51.6%). These two crops accounted for 7.5% of fruit production in 1986/90 and by 2002/06 accounted for 22.3% of production.

BC is a major fruit producer in Canada, second only to Ontario [23]. Blueberry production has skyrocketed since 1986, from less than 5,000 tonnes per year to more than 30,000 tonnes in 2008. BC now produces one third of Canadian blueberries as harvested hectarage has more than tripled. Grape production has increased rapidly with 17,000 tonnes produced in 2008. Over 90% of grapes are used in the wine industry [24].

### 3.5. Change in the Production of Grains, Oilseeds, & Hay in BC, 1986–2006

Most of the province’s grain crops are grown in the Peace River valley located in the province’s north central region. In 1986 BC produced 757,200 tonnes of grains harvested on 220,356 hectares of land. In 2006, BC produced 717,700 tonnes of grains from 128,497 hectares. Thus, during this 20 year period, production of grain decreased by about 5 percent and the hectarage of land planted with grain decreased by 42 percent.

Table 6 summarizes changes at the beginning and end of the study period showing that major declines in wheat, oats, and especially barley production have been balanced by increases (or stabilization) of corn grown for animal feed. Corn grown for animal feed represented just under half of grain production in 1986 and by 2006 it accounted for about two thirds of grain production.

### 3.6. Changes in Provincial Food Self-sufficiency, 1986–2006

As noted in our methods section and reiterated here, we have calculated self-sufficiency for each major food category by comparing estimates of consumption of each of these foods with estimates of production. Table 7 compares provincial food self-sufficiency in 1986 and 2006. It’s important to note that self-sufficiency could change over time because of changes in the amounts of food produced and/or because of some combination of changes in the amounts of various foods that people consumed over time and population growth. We have disentangled these potentially confounding trends by using estimates of production and consumption from 1986 in order to calculate food self-sufficiency for that year and, by using estimates of production and consumption from 2006 to calculate food self-sufficiency in 2006. In other words, we have taken account, by using consumption figures from 1986 and 2006 of the contribution of changes in consumption patterns (at least in the national population and assuming that they will apply to BC) to changes in self-sufficiency. This means that changes seen in Table 7 in self-sufficiency are due to changes in production and population growth alone.

Table 7 indicates that provincial self-sufficiency has dropped from 72 to 49% for fruit, from 39 to 13% for non-potato field vegetables, and from 267 to 54% for cereals mostly destined for human rather than animal consumption. Greenhouse vegetables have not been included in this table as most that are produced in BC are destined for export. (If we were to add all greenhouse vegetable production in 2006 to field vegetable production and, if we assumed that all greenhouse vegetables produced in BC were also consumed in the province, this would still demonstrate only 52% self-sufficiency in vegetable production (excluding potatoes) in 2006).

In contrast, meat self-sufficiency has increased from 64 to 94% and fluid milk self-sufficiency from 231 to 251 %. While egg self-sufficiency has dropped somewhat in BC between 1986 and 2006, it still remains well above 100%. Self-sufficiency for potatoes in BC has remained above 85% in these two years.

## 4. Discussion

Since the 1990s nutritionists in most developed nations have actively encouraged people to eat more vegetables, fruits, and whole grains. The question this paper seeks to address is to what extent have changes in the basic infrastructure of food production in one region, in this case the Canadian province of British Columbia, occurred which increase the availability of grains for human consumption, vegetables, and fruit, and alternatively, to what extent are these trends in production leading to a decrease in the availability of foods that supply saturated fat to human diets.

The basic infrastructural changes in land use and agricultural production in BC have led to less devotion of land to, and production of, field grown vegetables (except potatoes), fruit, and grains meant mainly for human consumption. At the same time, the amount of land and production devoted to animals and animal products have increased, especially with regard to production of dairy and beef cattle and poultry and to the amount of land devoted to animal pasturage and the production of grain for animal feed. As well, potato production has shown a slow but steady growth during this time. (However, roughly half of potato production in BC is processed into frozen and fast fry potatoes making them less nutritional and healthful [25].

While these are the general trends observable in the province over a 20 year period, the more specific trends indicate massive production growth in a few specialty green house vegetables destined mainly for export markets and, in the case of fruit, significant expansion of blueberry production capacity and considerable growth in grape production for wine. At the same time, there has been a major decline in the production of staple fruits (especially apples, peaches and pears), which have been produced in BC since the 19th century.

It is clear that even if “local” production of grains for human consumption, vegetables, and fruits decline these can and are supplemented by imports of these from markets in other parts of Canada and from other nations. However, it is also important to empirically determine changes in production that are local because these types of changes may also be underway in other jurisdictions and may act therefore, to the extent that they are underway, to decrease global availability of these types of healthy foods.

As well as examining trends in food production by major food category over time we have also examined these in relation to changes in population and consumption demand. This analysis indicates that in 1986 non-potato field vegetable production met about 40% of the British Columbian population’s consumption demand but, by 2006, only 13% of this demand was being met locally. While one could argue that rapid expansion of greenhouse vegetable production had, by 2006, boosted self-sufficiency to about 50% (more than it was in 1986) because greenhouse production is focused on four specialty crops and, because most greenhouse production is exported, it is unclear how much the expansion of this industry has contributed to enhanced provincial self-sufficiency in vegetables.

Considering fruit production we note similar trends as, in this case, two specialty fruits, grapes and blueberries, account for an increasing proportion of provincial fruit production and, as in the case of greenhouse vegetables, a considerable portion of these crops are destined for export markets. Thus, both fruit and vegetables, production, (in fields and orchards) of historically important species has declined markedly in the province as these sectors have generated new capacity in specialized production with a greater focus on export markets. These changes must be viewed in the context of greater overall agricultural focus, by 2006 in comparison to in 1986, on animal production both in terms of amount of land devoted to grain for animal relative to human feeding and, in terms of the direct production of animals.

There are complex reasons for the changes in the scope and intensity of food production in BC over this study period. These include, but are not limited to, rapid increase in the price of agricultural land, especially in the South-west region of the province, rapid increase in the price of basic agricultural inputs such as oil and fertilizer, globalization of food distribution and retailing, competing pressures on agricultural land (especially prime fruit and vegetable growing land in the South-west of the province and in the Okanagan valley) for other uses, and finally, changes in the economies of scale and the economics of production with major food trading partners. In turn, these complex changes have been influenced by relatively unpredictable events such as avian flu scares, 21st century outbreaks of “mad cow” disease, and widely publicized listeriosis poisoning originating in a major meat processing facility in Ontario. These more unpredictable food-safety related events have acted, arguably, to strengthen the economic hand of large food producers (who while they might operate locally tend to be provincially, nationally or inter-nationally focused) especially over the past decade, relative to small more local producers.

There are, however, some countervailing pressures in British Columbia that are being supported by local government, the provincial government and by some Regional Health Authorities. Just as food safety authorities and many food production systems are moving to more centralized control, consumption of local foods has become a popular issue perhaps with enough economic clout (as witnessed by the rapid expansion of local farmers’ markets in the province and the price premium many, mainly more well-heeled consumers, are prepared to pay for local produce), to begin to shift the momentum of these trends in local food production in a more favorable direction.

There are greater environmental and public health consequences of allowing these trends away from local production to continue. In particular, as climate change evolves industrial food production methods which rely on intensive fertilization and other oil-based inputs will be less tenable than they are now. And, food production and transportation systems that produce high emissions of greenhouse gas will face increasing scrutiny. Older more local methods of producing vegetables may, for example, be more suited for agricultural systems seeking to adapt in a world where reducing the carbon footprint of agricultural operations may trump other issues of agricultural policy.

Finally, focusing on the public heath dimension, the reduction of chronic diet-related illness in large populations is linked intimately to availability, price, and geographic access, especially to those foods which nutritionists recommend and that people currently under consume. These foods are mainly, but not exclusively, whole grains, vegetables, and fruit. Populations that do not have reasonably local access to these foods produced in an environmentally sustainable manner, may not be able, especially as climate change evolves, to access these in the quantity and quality recommended by nutritionists limiting our ability to decreases the prevalence of diet-related chronic ill health.

## 5. Conclusions

A health and nutritional assessment of the fundamental changes in BC agriculture between 1986 and 2006 indicates a structural shift in production and production systems towards lowered provincial self-sufficiency in those foods that nutritionists recommend we consume in greater quantities. Furthermore, exacerbating the structural disconnect, these shifts have led to self-sufficiency in foods that nutritionists indicate are at present either consumed in adequate amounts or that are over consumed.

## Figures and Tables

**Table 1.** Hectares of Farmland in BC Planted with Various Crops, 1986–2006.

	**1986**		**2006**		**20-Year Change (%)**	
	ha	% total	ha	% total	ha	% difference
**Total farmland**	**2,411,060**		**2,835,458**		**424,398**	**18**
Land in crops	570,843	24	586,238	21	15,395	3
*Total fruit*	*17,984*	*0.7*	*19,822*	*0.7*	*1,838*	*10*
*Total field vegetables^a^*	*7,569*	*0.3*	*6,957*	*0.2*	*−612*	*−8*
*Total Potatoes*	*3,182*	*0.1*	*3,439*	*0.1*	*257*	*8*
*Total grain*	*220,356*	*9.1*	*128,497*	*4.5*	*−91,859*	*−42*
*Total hay*	*…*		*391,595*	*14*	*…*	*…*
Summer fallow land	81,167	3.4	25,581	0.9	−55,586	−68
Pastureland	1,239,813	51	1,745,356	62	505,543	41
All other land	519,238	22	478,283	17	−40,955	−8
*Suppressed data*	*321,752*	*13*	*35,928*	*1.3*	*−285,824*	*−89*
Nursery products^b^	1,566		4,505		2,939	188
Greenhouse vegetables^c^	1,306,123	100	5,293,197	100	3,987,074	305

Data source: Statistics Canada Census of Agriculture, 1986–2006;

Some census data is suppressed to meet confidentiality requirements of *Statistics Act*;

a Does not include potatoes;

b Includes Christmas tree land, woodlands, and wetlands;

c Not included in total farmland values; greenhouse vegetable area expressed in square meters covered by greenhouse glass.

**Table 2.** Number of Livestock Animals in BC, 1986–2006.

	**1986** (% total)	**2006** (% total)	**20-year Change** (%)
Beef cattle and calves (non-dairy)	614,952 (64)	728,099 (73)	113,147 (+18)
Dairy cows	75,005 (8)	72,756 (7)	−2,249 (−3)
Pigs and hogs	216,732 (22)	135,826 (14)	−80,906 (−37)
Sheep and lambs	57,243 (6)	61,033 (6)	3,790 (+7)
**Total Number of Animals**	**963,932 (100)**	**997,714 (100)**	**33,782 (+3)**
**Total poultry**	**10,243,807 (100)**	**20,446,080 (100)**	**10,202,273 (+100)**

Data source: Statistics Canada Census of Agriculture, 1986–2006.

**Table 3.** Production of Dairy & Eggs in BC, 1986–2006.

	**1986**	**2006**	**20–year Change (%)**
Milk Production (kilolitres)	488,838	627,235	138,397 (+28%)
Milk Yield (l/dairy cow)	6,517	8,621	2,104 (+32%)
Egg Production (doz)	54,761	93,612	38,851 (+71%)
Egg Yield (eggs/hen)	255.1	244.4	11 (−4%).

Data source: Statistics Canada CANSIM Tables 003–0011 & 003–0020.

**Table 4.** Summary Statistics, Changes in BC Vegetable Production, 1986–2006.

	1986–1990 Mean	2002–2006 Mean	% diff.
Potato	84,121	110,758	+32
Other Field Veg	79,455	47,820	−40
Greenhouse Veg^a^	20,036	107,531	+437
**Total**	**183,612**	**266,109**	

a. Data for greenhouse vegetables not available until 1993;

mean for 1993–95 used instead of mean from 1986–1990.

**Table 5.** Summary Statistics, Changes in BC Fruit Production, 1986–2006.

	1986–1990 Mean	2002–2006 Mean	% diff.
Apples	173,259	113,200	−34.7
Peaches	8,390	5,371	−36
Pears	8,530	5,862	−31.3
Plums & prunes	1,989	986	−50.4
Raspberries	16,600	11,251	−32.2
Strawberries	6,477	2,787	−57
Blueberries	7,056	24,345	+245
Grapes	10,307	15,624	+51.6
**Total**	**232,608**	**179,427**	−22.9

Data source: Statistics Canada CANSIM Table 001–0009.

**Table 6.** Summary Statistics, Changes in BC Grain Production, 1986–2006.

	1986–1990 Mean	2002–2006 Mean	% diff.
All wheat	110,400	47,860	−56.7
Oats	67,220	53,400	−20.6
Barley	138,040	91,900	−33.4
All rye	7,860	220	−97.2
Mixed grains	5,360	4,720	−12
Dry field peas		4,900	
Canola	39,480	38,240	−3.1
Fodder corn	433,640	463,560	6.9
**Total**	**802,000**	**704,800**	**−12.1**

Data source: Statistics Canada CANSIM Table 001–0017.

**Table 7.** Provincial Consumption and Production of Food, 1986 & 2006 and % Self-Sufficient.

	**Provincial Consumption^a^**		**Provincial Production**		**% Self-sufficient**	
*tonnes*	**1986**	**2006**	**1986**	**2006**	**1986**	**2006**
Total fruits	233,231	388,075	168,335	188,879	72%	49%
Potatoes	105,968	121,282	91,000	108,182	86%	89%
Field vegetables^b^	193,253	308,805	76,043	39,049	39%	13%
Cereal human conspt.^c^	141,050	245,746	376,200	132,600	267%	54%
Meat^d^	204,967	276,257	131,688	259,245	64%	94%
Fluid milk (kilolitres)	211,275	249,565	488,808	627,229	231%	251%
Eggs ('000 doz)	39,287	51,602	58,987	63,370	150%	123%

Data Sources: CANSIM Table 051–0001 (population estimates); CANSIM Table 002–0019 (per-capita consumption estimates); 2006 Census of Agriculture (# laying hens, livestock animals, dairy cows);

CANSIM Tables 003–0011, 003–0020, 003–0035, & 003–0036 (estimated yields per animal);

a Provincial consumption estimated through National Disappearance Data (providing per-capita consumption estimates as part of “Food Statistics” Statistics Canada annual publication), multiplied by annual provincial population;

b Total vegetables excludes potatoes, which are presented separately and excludes greenhouse vegetables. These latter are mainly grown for export so are excluded from this table;

c Cereal products excludes corn for forage but includes all others (wheat, barley, canola, rye, mixed grains, oats) and so roughly describes cereals grown for human consumption;

d Total meat includes red meat (beef, pork, lamb) and poultry (turkey, chicken); 1991 meat yields used in lieu of unavailable 1986 meat yields.
